# The Biosynthetic Pathways of Tanshinones and Phenolic Acids in *Salvia miltiorrhiza*

**DOI:** 10.3390/molecules200916235

**Published:** 2015-09-08

**Authors:** Xiao-Hui Ma, Ying Ma, Jin-Fu Tang, Ya-Li He, Yu-Chen Liu, Xiao-Jing Ma, Ye Shen, Guang-Hong Cui, Hui-Xin Lin, Qi-Xian Rong, Juan Guo, Lu-Qi Huang

**Affiliations:** 1 Pharmacy College, Chengdu University of Traditional Chinese Medicine, Chengdu 611137, China; E-Mail: maxiaohui1988@126.com; 2 State Key Laboratory Breeding Base of Dao-Di Herbs, National Resource Center for Chinese Materia Medica, China Academy of Chinese Medical Sciences, Beijing 100700, China; E-Mails: xiaoma1110@126.com (Y.M.); jinfutang@126.com (J.-F.T.); yalichn@126.com (Y.-L.H.); lyc8564732@163.com (Y.-C.L.); maxiaojing.ok@163.com (X.-J.M.); shenye70@hotmail.com (Y.S.); guanghongcui@163.com (G.-H.C.); lhxbio@126.com (H.-X.L.); rqx1982@126.com (Q.-X.R.); 3 Beijing Key Laboratory of Protection and Application of Chinese Medicinal Resources, Beijing Normal University, Beijing 100875, China; 4 Pharmaceutical College, Guiyang College of Traditional Chinese Medicine, Guiyang 550002, China

**Keywords:** tanshinones, phenolic acids, *Salvia miltiorrhiza*, biosynthetic pathway, metabolic engineering

## Abstract

Secondary metabolites from plants play key roles in human medicine and chemical industries. Due to limited accumulation of secondary metabolites in plants and their important roles, characterization of key enzymes involved in biosynthetic pathway will enable metabolic engineering or synthetic biology to improve or produce the compounds in plants or microorganisms, which provides an alternative for production of these valuable compounds. *Salvia miltiorrhiza*, containing tanshinones and phenolic acids as its active compounds, has been widely used for the treatment of cardiovascular and cerebrovascular diseases. The biosynthetic analysis of secondary metabolites in *S. miltiorrhiza* has made great progress due to the successful genetic transformation system, simplified hairy roots system, and high-throughput sequencing. The cloned genes in *S. miltiorrhiza* had provided references for functional characterization of the post-modification steps involved in biosynthesis of tanshinones and phenolic acids, and further utilization of these steps in metabolic engineering. The strategies used in these studies could provide solid foundation for elucidation of biosynthetic pathways of diterpenoids and phenolic acids in other species. The present review systematically summarizes recent advances in biosynthetic pathway analysis of tanshinones and phenolic acids as well as synthetic biology and metabolic engineering applications of the rate-limiting genes involved in the secondary metabolism in *S. miltiorrhiza*.

## 1. Introduction

Plant secondary metabolites including terpenes, phenolic compounds, and nitrogen-containing compounds, which are served as signaling or defense molecules, are not only important to plant development and adaptation, but also have significant economic value as resources in human medicine and chemical industries. Many secondary metabolites from plants have been used in the clinical treatment of diseases, such as taxol in the treatment of refractory ovarian cancer and metastatic breast cancer [1], and ginkgolides in the treatment of cardiovascular diseases and diabetic vascular complications [2]. Secondary metabolites are always accumulated at low levels in plants with long growth periods. Moreover, chemical synthesis is challenging and has little practical value for those compounds with complicated structures. Scientists have turned to biotechnology and genetic engineering to produce or improve these compounds in plants or microorgnisms.

Elucidation of the biosynthetic pathways of secondary metabolites in plants enabled the exploration of metabolic engineering or synthetic biology as potentially effective approaches for producing specific metabolites, which provide alternative for the production of the valuable compounds. Metabolic engineering can be optimized by enhancing the rate-limiting steps or blocking competitive pathways to improve production. Overexpression of genes that encode the rate-limiting enzymes involved in scopolamine biosynthesis has resulted in greater accumulation of scopolamine in hairy roots of transgenic *Hyoscyamus niger* compared to the wild-type [3]. Contents of oleanane-type ginsenoside (ginsenoside Ro) in *Panax ginseng* plant were highly increased by overexpression of *CYP716A52v2*, and the amount of ginsenoside Ro declined in an RNA interference transgenic line targeting this gene [4]. Alternatively, synthetic biology can be used to construct engineered strains for the high-yield production of bioactive secondary metabolites. Paddon *et al.* used synthetic biology to develop strains of *Saccharomyces cerevisiae* for the high-yield biological production of artemisinic acid, which led to the heterologous production of 25 g/L of this compound by baker’s yeast after multiple steps of optimization [5].

The root and rhizome of *S. miltiorrhiza* with diterpenoid tanshinones and phenolic acids as its active compounds is a traditional Chinese medicine (usually called *danshen* in China) has been widely used for the treatment of cardiovascular and cerebrovascular diseases. The Fufang Danshen Dripping Pill with *danshen* as its main material has been extensively used for treatment of cardiovascular diseases in China and some other countries, and has been approved for phase III clinical trials in the USA (ClinicalTrials.gov Identifier: NCT01659580). The two typical secondary metabolites made *S. miltiorrhiza* as a model for analyzing plant secondary metabolism pathways. Tanshinones including tanshinone I, tanshinone IIA, cryptotanshinone, and dihydrotanshinone I are lipophilic compounds, which are widely accumulated in *Salvia* (Labiaceae) [6,7]. More than 40 tanshinones have been isolated from *danshen*. It possesses significant antioxidant, anti-inflammatory, and antineoplastic activities and has been shown to have potent anticancer activities both *in vitro* and *in vivo* [8,9]. The anti-inflammatory activity of tanshinone IIA is especially outstanding, as this compound blocks pro-inflammatory signals *in vivo* and these effects are conserved in human neutrophils [10]. In addition, the sulfotanshinone sodium injection constituted with sodium tanshinone IIA sulfate, which significantly improves the intestinal absorption of it, has been widely used for treatment of coronary disease, angina pectoris, and myocardial infarction in China [11]. Tanshinones also have significant anti-aging activity. For instance, cryptotanshinone can greatly extend the chronological lifespan of budding yeast *S. cerevisiae* [12]. Phenolic acids are widely distributed in the plant kingdom, especially in the Boraginaceae and Labiaceae families [13]. These compounds include caffeic acid monomers as well as oligomers of danshensu, caffeic acid, salvianolic acids, rosmarinic acid (RA), and lithospermic acids [14]. Among more than 20 phenolic acids isolated from *danshen*, salvianolic acid B (SAB) is the major water-soluble constituent with particular activity, and the content of SAB in *S. miltiorrhiza* is more abundant than in other Labiaceae species [15]. Phenolic acids from *danshen* were prominent with its antioxidant, anticoagulant, and cell protection activities [14]. The main effective ingredients of Danhong Injection (China food and drug administration: Z20026866) are the water-soluble constituents, which have been used for treatment of various kinds of ischemic disease in China.

Due to the medicinal and economic value of these secondary metabolites in *danshen*, it is important to enhance the production of these compounds through metabolic engineering or synthetic biology. Elucidating the biosynthesis of these compounds and identifying the exact genes that catalyze their synthesis are critical for improving the production of these compounds. Fortunately, advances in sequencing and comparative transcriptome analysis have provided efficient candidates for biosynthetic pathway analysis. The successful hairy root transformation and generation system in *S. miltiorrhiza* have provided a simple system for studying the biochemical properties and gene functions [16,17,18]. Though the downstream biosynthetic pathway analysis of tanshinones and phenolic acids is still ongoing, it has made great progress in elucidating the biosynthesis of these secondary metabolites which could provide a solid foundation for other compounds. In this article, we systematically reviewed the recent progresses in biosynthetic pathway analysis of tanshinones and phenolic acids as well as synthetic biology and metabolic engineering applications of the rate-limiting genes involved in the secondary metabolism in *S. miltiorrhiza* from year 2005 to year 2015, and further discussed the analysis and utilization of the metabolic pathways.

## 2. Comparative Transcriptome Provides Informative Data in Biosynthetic Pathway Analysis

Advances in sequencing and bioinformatics analysis could provide a large amount of candidate genes for the biosynthetic pathway responsible for the secondary metabolites. *S. miltiorrhiza* hairy root could be generated by infecting leaf explants with *Agrobacterium rhizogenes* strains in *S. miltiorrhiza* [19,20] and exhibits a similar protective activity against hypoxia and reoxygenation injury in rat cardiac myocytes to *danshen* [21]. In addition, the secondary metabolites in hairy roots could be stimulated by elicitors within several days, which provide a simplified system to identity new candidate genes by different expression or comparative transcriptome analysis. The first cDNA microarray analysis for different stages of *S. miltiorrhiza* hairy root cultures revealed 203 differentially expressed genes from 4354 cDNA clones, including seven genes (especially two important terpenoid synthases) involved in tanshinone biosynthesis [22]. However, the information in cDNA microarray is limited, a large amount of candidates related to the post-modification progress in biosynthesis of secondary metabolites are to be investigated.

Deep sequencing could provide high-throughput data for the discovery of candidate genes involved in the biosynthesis of natural products, especially in non-model species. Furthermore, comparative transcriptome analyses of different tissues with and without active ingredients could provide a comprehensive insight into the biosynthesis and regulation of these compounds. In the transcriptome over the entire growth cycle, including the seedling stage, vegetable stage, and different tissues in the reproductive stage, a total of 47 unigenes encoding more than 24 enzymes and 200 unigenes related to 32 enzymes were identified, which are potentially involved in the biosynthesis of rosmarinic acids and terpenoids, respectively [23]. In a more recent study, Yang *et al.* further conducted transcriptomic profiling of the *S. miltiorrhiza* root and leaf tissues by using 454 GS-FLX pyrosequencing platform to identify genes and transcriptional regulators involved in the biosynthesis of tanshinones. 168 unigenes involved in the biosynthesis of the terpenoid backbone and 2863 unigenes that are highly expressed in roots were identified, including those genes involved in the biosynthesis of tanshinones, such as copalyl diphosphate synthase (*SmCPS1*), kaurene synthase-like (*SmKSL1*) and *CYP76AH1* [24].

The transcriptome among different tissues including different expression genes related to plant development resulted in too many candidates for further characterization, while the comparative transcriptome from different elicitation processes could significantly narrow the candidates for functional characterization. The transcriptional profiles of MJ elicitated *S. miltiorrhiza* leaves yielded 37647 unique sequences. Almost all of the known genes involved in tanshinone and phenolic acid biosynthesis in *S. miltiorrhiza* were expressed differentially between the untreated and treated samples. Three and four candidate P450s that could be involved in the biosynthesis of tanshinones and phenolic acids, respectively, were selected from the RNA-seq data based on the co-expression analyses with *SmCPS1*/*SmKSL1* or *SmRAS* [25]. Metabolomics could further provide an instantaneous snapshot of the entire physiology of an organism by comparing the relative differences between biological samples based on their metabolite profiles. Gao *et al.* combined metabolomics and deep sequencing to investigate the inducible biosynthesis of tanshinones. The comprehensive transcriptome comprised 20972 non-redundant genes, of which 39 CYPs exhibited up-regulation at all time points following induction. Eight cytochrome P450s (P450s) were co-regulated with known genes involved in tanshinones biosynthesis, providing targets for future investigations [26]. This study provided reference for combining metabolomics and deep sequencing to investigate new targets for future secondary metabolic pathway analysis in *S. miltiorrhiza*.

P450s have been demonstrated to play key role in post-modification of tanshinones biosynthesis [27]. Using RNA-Seq technology, Chen *et al*. systematically studied the P450 genes in *S. miltiorrhiza*, and identified 116 full-length and 135 partial-length P450 genes by combining their data with previously identified P450 sequences and scanning the data with the P450 model from Pfam [28]. Through a comparison with the KEGG database, ten of these P450 genes were speculated to be related to diterpenoid biosynthesis, three genes were predicted to be potentially involved in terpenoid biosynthesis. The database set up in this study provided a centralized resource for P450 exploration in secondary metabolism analysis in *S. miltiorrhiza*. More recently, combination of next-generation sequencing (NGS) and single-molecule real-time (SMRT) sequencing was applied to various root tissues to generate a much more complete transcriptome of *S. miltiorrhiza*, particularly including the periderm, where the biosynthesis and accumulation of tanshinones occurred. Combining this transcriptome data with qRT-PCR to analyze these genes expression pattern, 15 CYPs, one 2-oxo-glutarate dependent di-oxygenase (2ODD) and five short-chain alcohol dehydrogenases (SDRs) which co-expressed with *SmCPS1*, *SmKSL1*, and *CYP76AH1* were predicted to play roles in tanshinones biosynthesis [29]. These databases provide valuable resource and full-length candidate genes for further investigation of secondary metabolites biosynthesis in *S. miltiorrhiza*.

## 3. Biosynthesis of Tanshinones in *S. miltiorrhiza*

**Figure 1:**
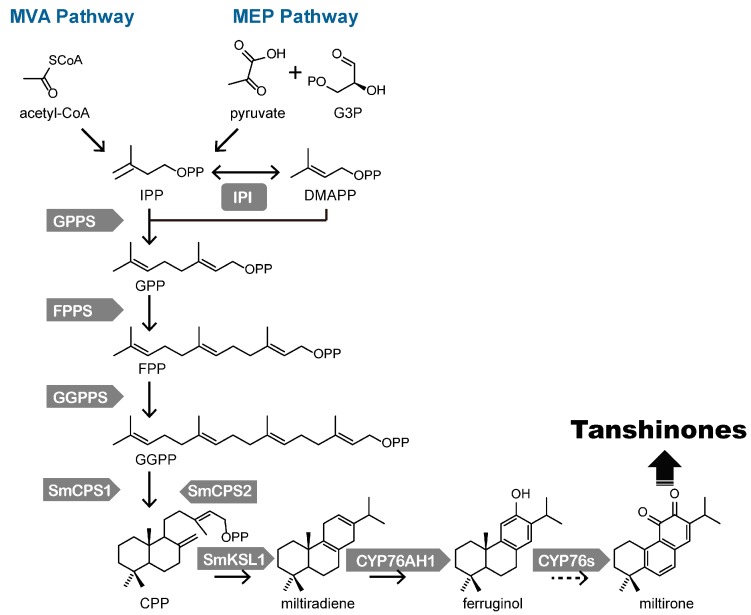
Biosynthethsis of tanshinones in *S. miltiorrhiza*. Solid arrows indicate the established relationships, and dashed arrows indicate hypothetical relationships. G3P: glyceraldehyde-3-phosphate, IPP: isopentenyl pyrophosphate, DMAPP: dimethylallyl pyrophosphate, IPI: IPP isomerase, GPP: geranyl diphosphate, GPPS: GPP synthase, FPP: farnesyl diphosphate, FPPS: FPP synthase, GGPP: geranylgeranyl diphosphate, GGPPS: GGPP synthase, CPP: copalyl diphosphate, SmCPS: CPP synthase, SmKSL: kaurene synthase-like cyclase.

Tanshinones are a group of abietane-type norditerpenoid quinone natural products that primarily accumulate in the roots of *S. miltiorrhiza*. The biosynthetic pathway of tanshinones could be divided into three steps: the formation of precursors for all terpenoids; the construction of skeletons of tanshinones; and the post-modification of the skeleton, such as oxidation, methylation, decarboxylation or cyclization to produce the various tanshinones. This pathway has been explored for more than two decades, and most genes involved in tanshinone biosynthesis have been cloned (Table 1 and Figure 1) [6,26,27].

**Table 1:** The genes involved in isoprenoids metabolic pathway in *S. miltiorrhiza*.

Gene (Genbank No.)	Highest Expression Sites	Elicitor	Reference
*SmAACT1* (EF635969)	root	YE, Ag^+^	[30]
*SmAACT2* (AEZ55671)	stem		[31]
*SmHMGS* (FJ785326)	leaf	SA, MJ, YE	[32]
*SmHMGR1* (EU680958 GU367911)	flower and root	SA, JA, MJ, PEG, ABA	[31,33,34]
*SmHMGR2* (FJ747636)	leaf, stem, root	MJ, Ag^+^	[22,31,35,36]
*SmHMGR3* (JN831102)	leaf	MJ	[31,37]
*SmHMGR4* (JN831103)	flower		[31]
*SmMK* (JN831104)	stem	MJ	[31]
*SmPMK* (JN831095)	stem and root	MJ	[31]
*SmMDC* (JN831105)	stem and root	MJ	[31]
*SmDXS1* (EU670744)	leaf	Ag^+^	[31,35]
*SmDXS2* (FJ643618)	root and leaf	MJ, PEG, ABA, MJ Act12, Ag^+^	[17,31,34,35,38]
*SmDXS3* (JN831116)	leaf, stem, root cortex, and root stele	MJ	[31]
*SmDXS4* (JN831117)	ubiquitous		[31]
*SmDXS5* (JN831118)	leaf and stem		[31]
*SmDXR* (FJ768959, FJ476255, DQ991431)	leaf	SA,YE, Hypertonic stress, Act12 PEG, ABA and MJ, Ag^+^	[34,35,38,39,40]
*SmCMT* (JN831096)	leaf		[31]
*SmCMK* (EF534309)	leaf	MJ	[31,41]
*SmMCS* (JX233816)	leaf and stem	Ag^+^	[31,42]
*SmHDS1* (JN831098)	leaf		[31,43]
*SmHDS2* (KJ746807)			[44]
*SmHDR1* (JX233817 JN831099)	leaf	MJ, Ag^+^	[31]
*SmHDR2* (JN831100)	leaf		[31]
*SmHDR* (JX516088)	leaf	MJ, SA	[45]
*SmIPI1* (JN831106)	root	MJ, fungal elicitor	[22,46]
*SmIPI2* (JN831106)	stem	MJ	[31]
*SmGPPS* (JN831107)			[31]
*SmGPPS*.LSU (JN831111)	stem and flower	MJ	[31]
*SmGPPS*.SSUI (JN831108)	leaf	MJ	[31]
*SmGPPS*.SSUII.1 (JN831109)	leaf and root cortex		[31]
*SmGPPS*.SSUII.2 (JN831110)	stem		[31]
*SmFPPS* (EF635968 HQ687768)	stem	MJ	[22,31,47]
*SmGGPPS1* (FJ643617 FJ178784)	leaf	NaCl, wounding, high temperature, darkness, pathogen, MJ, ABA, SA and GA, Act12, Ag^+^	[17,35,38,48,49,50]	
*SmGGPPS2* (JN831112)	root stele		[31]
*SmGGPPS3* (JN831113)	ubiquitous		[31]
*SmCPS1* (EU003997)	root cortex	Ag^+^, MJ, YE	[35,51,52]
*SmCPS2* (JN831114)	leaf		[31,53]
*SmCPS3* (JN831115)	stem		[31,53]
*SmCPS4* (JN831120)	sepal		[31,53]
*SmCPS5* (JN831121)	stem	MJ	[31,53]
*SmKSL1* (EF635966)	root	Ag^+^, MJ	[35,51,53]
*SmKSL2* (JN831119)	root xylem		[31,53]
*CYP76AH1* (JX422213)	root	Ag^+^	[27,35]
*CYP76s* (KR140168) (KR140169)	root	Ag^+^	[54]
*SmCPR1*(FR693803)			[27,55]
*SmCPR2*(JX848592)			[27]

Isopentenyl diphosphate (IPP) and its isomer dimethylallyl diphosphate (DMAPP) are the precursors of all terpenoids. In plants, two biosynthetic pathways are involved in the production of the universal isoprene precursors IPP and DMAPP: the mevalonate (MVA) pathway in the cytosol, and the methylerythritol phosphate (MEP) pathway in the plastids. The MEP pathway was identified through labeling experiments in bacteria and plants [56,57]. In plants, the MEP pathway is mainly responsible for production of monoterpenoids, diterpenoids, carotenoids, and the side chains of chlorophylls and plastoquinone [58]. Glyceraldehyde 3-phosphate (G3P) and pyruvate have been identified as the direct precursors of IPP and DMAPP. Most genes in the MEP pathway have been identified in *S. miltiorrhiza.* 1-Deoxy-d-xylulose-5-phosphate synthase (DXS) is the key enzymes of the MEP pathway and is involved in the important rate-limiting reaction. There are five DXSs in the draft genome of *S. miltiorrhiza*, and the expression level of *SmDXS2* in *S. miltiorrhiza* roots was highest compared with the other *SmDXSs* [31]. It was also reported that the overexpression of *SmDXS2* could increase the accumulation of tanshinones in *S. miltiorrhiza* hairy root [17], indicating that *SmDXS2* might be closely related to the accumulation of tanshinones.

The MVA pathway, which has been studied for decades, is maily involved in the biosynthesis of sesquiterpenoids, polyisoprenoids and sterols [59]. In *S. miltiorrhiza*, it was considered that tanshinones are mainly synthesized by the MEP pathway, while the MVA pathway could be better explained as being involved in cell growth. However, both cell growth and tanshinone production could partially depend on the crosstalk between the two pathways [60,61]. In the MVA pathway, IPP is synthesized from two acetyl-CoA molecules. The 3-hydroxy-3-methylglutaryl-CoA reductase (HMGR) converts 3-hydroxy-3-methylglutaryl-CoA (HMG-CoA) to MVA with two NADPH as the rate-limiting step of the MVA pathway [36]. There are four HMGRs in the draft genome of *S. miltiorrhiza* [31]. *SmHMGR1*, *SmHMGR2*, *SmHMGR3* are considered to play more important roles in tanshinone biosynthesis [31]. Actually, overexpression of *SmHMGR1* [17,62] and *SmHMGR2* [36] in *S. miltiorrhiza* hairy root cultures both could significantly increase the tanshinone content, indicating that *SmHMGR1* and *SmHMGR2* from MVA pathway might also contribute to the biosynthesis of tanshinones.

The conversion of IPP and DMAPP could be catalyzed by IPP isomerase (IPI). DMAPP and IPP are further condensed by geranyl diphosphate synthase (GPPS), farnesyl diphosphate synthase (FPPS), geranylgeranyl diphosphate synthase (GGPPS) to form GGPP [6]. GGPP is the universal precursor of all diterpenoids and can be catalyzed by different terpenoid synthases to form various diterpenoid skeletons. Terpenoid synthases are the key enzymes for the biosynthesis of the tanshinone skeletons. 12 *SmCPS* and nine *SmKSL* homologs have been reported in *S. miltiorrhiza* [29]. The functions of four *SmCPSs* and two *SmKSLs* were identified through the expression of the protein in *Escherichia coli* and enzymatic catalysis *in vitro* combined with GC-MS measurements, which characterized the role of *SmCPS1*, *SmCPS2*, and *SmKSL1* in tanshinone biosynthesis to form the skeleton miltiradiene [51,53], and kenetic analysis showed that *SmCPS1* had both higher affinity and activity with GGPP than *SmCPS2*. RNA interference was employed to down-regulate the expression of *SmCPS1*, which resulted in a decrease in the accumulation of tanshinones, further verified the key role of *SmCPS1* in tanshinone biosynthesis [16,53].

According to the structure of tanshinones, the genes involved in the downstream pathway involved in tanshinone biosynthesis are P450s, dehydrogenases, demethylases, and other structural modifying enzymes, which always begin with P450s [27,63]. Guo *et al.* [27] used comparative transcriptome analysis to identify six candidate P450s genes that were co-regulated with the diterpenoid synthase genes in both the root and *S. miltiorrhiza* hairy roots. *CYP76AH1* was identified to catalyze miltiradiene to produce ferruginol both *in vitro* and *in vivo*. Recently, based on co-expression analysis, the authors further identified two new P450s which can work sequentially to catalyze ferruginol into the metabolic intermediate, which with two keto in C11 and C12 is more close to the tanshinones [54]. Now, the post-modification from ferruginol to tanshinones is still ongoing. The identified enzymes involved in biosynthetic pathway could provide reference genes for post-modification characterization.

## 4. Biosynthesis of Phenolic Acids in *S. miltiorrhiza*

There are more than 20 phenolic acids in *S. miltiorrhiza*, with RA and SAB as the typical water-soluble constituents which contribute to the effects in the treatment of cardiovascular disease. RA was a dominant phenolic acid distributing in Lamiaceae. The biosynthetic pathway of RA in *Coleus blumei* has been characterized [13]. In Lamiaceae including *S. miltiorrhiza*, two biosynthetic pathways are involved in the biosynthesis of the processor of phenolic acid: the phenylpropanoid pathway and the tyrosine-derived pathway. In the phenylpropanoid pathway, phenylalanine is transformed into 4-coumaroyl-CoA by phenylalanine ammonia-lyase (PAL), cinnamic acid 4-hydroxylase (C4H) and 4-coumarate: CoA ligase (4CL). In the tyrosine-derived pathway, tyrosine is metabolized to 4-hydroxyphenyllactate through tyrosine aminotransferase (TAT) and 4-hydroxyphenylpyruvate reductase (HPPR), finally forming 3,4-dihydroxyphenyllactic acid (DHPL, also known as salvianic acid A or danshensu). It was reported that the expression of enzymes involved in the tyrosine-derived pathway were more correlated to rosmarinic and salvianolic acid B biosynthesis after elicited with MJ, hyphal extracts or other elicitors, which indicated that the tyrosine-derived pathway might be the rate-limiting step in biosynthesis of phenolic acid [64,65]. 4-coumaroyl-CoA and DHPL were further coupled by the ester-forming enzyme rosmarinic acid synthase (RAS) to form 4-coumaroyl-3′4′-dihydroxyphenyllactic acid (4C-DHPL). 4C-DHPL is oxidized by *CYP98A14*, which introduces the 3-hydroxyl group to form RA.

**Figure 2:**
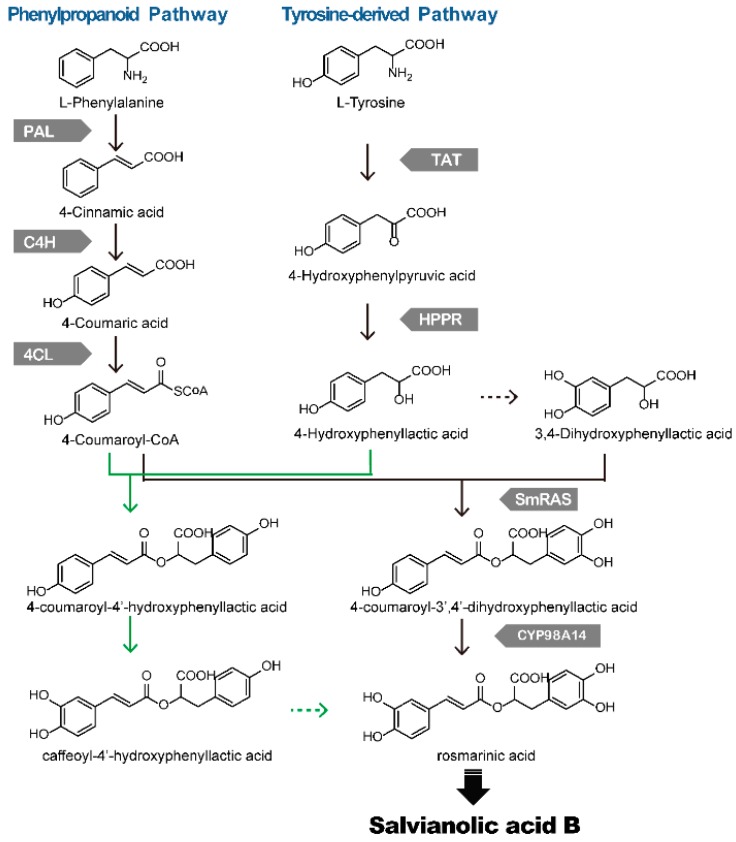
Biosynthethsis of phenolic acids in *S. miltiorrhiza* [15]. Solid arrows indicate the established relationships, and dashed arrows indicate hypothetical relationships. RA is mainly biosynthesized from the pathway showed with black arrows in *S. miltiorrhiza*, and part is from the pathway showed with green arrows. PAL: phenylalanine ammonialyase, C4H: cinnamic acid 4-hydroxylase, 4CL: 4-coumaroyl: CoA ligase, TAT: tyrosine aminotransferase, HPPR: 4-hydroxyphenylpyruvate reductase, RAS: rosmarinic acid synthase.

RA was believed to be the processor of SAB. Di *et al.* [15] employed isotopic labeling experiments *in vivo* using [ring-13C]-phenylalanine to trace the metabolic origin of phenolic acids in *S. miltiorrhiza.* It was speculated that RA might be transformed into a phenoxyl radical by oxidation, and two phenoxyl radicals unite spontaneously to form SAB [15]. In *S. miltiorrhiza*, 4C-DHPL was considered as the main intermediate for RA biosynthesis, and DHPL was involved in this pathway as a principal intermediate. However, the pathway from RA to LAB remains to be elucidated (Figure 2).

The majority of genes involved in phenolic acid biosynthesis have been cloned in *S. miltiorrhiza*, including *SmPAL*, *SmC4H*, *Sm4CL*, *SmTAT*, *SmHPPR*, *SmRAS* and *CYP98A14* (Table 2). Three *SmPALs* were identified in the working draft of the *S. miltiorrhiza* genome. Coexpression analyses with the accumulation of phenolic acids revealed that *SmPAL1* and *SmPAL3* were more abundant in the root which indicated that they might be more important in the biosynthesis of rosmarinic acids [66]. The function of *SmPAL1* was further verified by *in vivo* and *in vitro* analyses, which revealed that *SmPAL1* exhibited high catalyzation activity using L-Phe as substrate [67]. Otherwise, when the expression of *SmPAL1* was suppressed in *S. miltiorrhiza*, the rosmarinic acid and salvianolic acid B content was significantly decreased which proved the key role of *SmPAL1* in biosynthesis of phenolic acids [68]. Several Sm4CLs have been identified in *S. miltiorrhiza.* The enzyme activity analyses and expression analyses of *Sm4CL2* and *Sm4CL3* indicated that these two genes are more likely to be involved in the biosynthesis of water-soluble phenolic compounds [69,70,71]. In addition to PAL and 4CL, the function of *SmC4H1*, *SmTAT1*, and *SmHPPR1* were also confirmed *in vivo*. Overexpression in the hairy roots of *S. miltiorrhiza* could increase the accumulation of RA and SAB [18]. The functional characterization genes involved in the biosynthesis of phenolic acids could be used as targets in metabolic engineering to accelerate the production of these compounds.

**Table 2:** The genes involved in phenolic acids biosynthesis in *S. miltiorrhiza*.

Gene (Genbank No.)	Highest Expression Sites	Elicitor	Reference
*SmPAL1* (EF462460)	root, leaf	ABA, wounding and dehydration, PEG, MJ, SA, Ca^2+^, GA, ethylene	[66,67,68,71,72,73,74,75]
*SmPAL2* (GQ249111)	stem, flower	PEG and MJ	[66,68,71]
*SmPAL3* (KF220569)	rootleaf	PEG and MJ	[66,71]
*SmC4H1* (EF377337 DQ355979)	root, stem	MJ, ABA, UV-B, Ag^+^	[35,71,76,77]
*SmC4H2* (KF220564)	stem, root		[71]
*Sm4CL1* (AY237163)	leaf	MJ, YE	[69,70]
*Sm4CL2* (AY237164)	root	MJ, YE, Ag^+^	[35,69,70,71]
*Sm4CL3* (KF220556)	root	MJ	[71]
*Sm4CL-like1* (KF220557)	root	MJ	[71]
*Sm4CL-like2* (KF220558)		MJ	[71]
*Sm4CL-like3* (KF220559)			[71]
*Sm4CL-like4* (KF220560)	root		[71]
*Sm4CL-like5* (KF220561)			[71]
*Sm4CL-like6* (KF220562)		MJ	[71]
*Sm4CL-like7* (KF220563)			[71]
*SmTAT1* (DQ334606 EF192320)	stem	MJ, ABA, SA, UV-B, GA, ethylene, Ag^+^, YE	[35,65,72,74,78]
*SmTAT2* (KF220575)	flower	MJ	[71]
*SmTAT3* (KF220555)	Stem, root		[71]
*SmHPPR1* (DQ099741 DQ266514 EF458148)	stemflower	MJ, SA, GA3, ABA, UV-B, Ag^+^	[35,71,79]
*SmHPPR2* (KF220565)	Stem,leaf		[71]
*SmHPPR3* (KF220566)	stem		[71]
*SmHPPR4* (KF220567)			[80]
*SmRAS-Like* (GU647199)	stem	*Pseudomonas lachrymans*, MJ, light, and SA	[81]
*SmRAS1* (FJ906696)	root	MJ, Ag^+^	[15,35]
*SmHCT1* (KF220570)	root		[71]
*SmHCT2* (KF220571)	stem		[71]
*SmHCT3* (KF220572)	stem	MJ	[71]
*SmHCT4* (KF220573)	stem	MJ	[71]
*SmHCT5* (KF220574)	stem	MJ	[71]
*CYP98A14* (HQ316179)	root	MJ, Ag^+^	[15,35]

## 5. Biotechnological Applications

**Figure 3:**
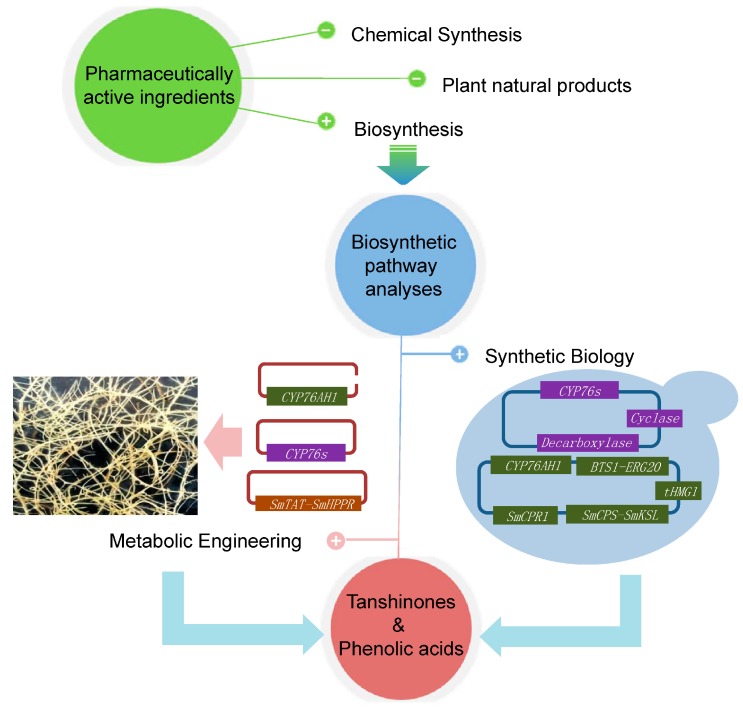
Biotechnogical applications in *Salvia miltiorrhiza*.

Natural plant products are often produced in relatively low amounts and are difficult to chemically synthesize. Biotechnology is one of the most important ways to improve the active compounds of plants. Elucidating the secondary metabolites biosynthetic pathway is helpful for further revealing the mechanisms that produce bioactive components. Based on the biosynthetic pathway analyses, synthetic biology and metabolic engineering have been used to produce or improve the production of tanshinone and phenolic acid in both microorganisms and *S. miltiorrhiza* or its hairy roots (Figure 3).

The advances in recombinant DNA technology offered the opportunity of using synthetic biology to produce tanshinones and phenolic acid-related compounds in microorganisms. Zhou *et al.* applied a modular pathway engineering (MOPE) strategy for the rapid assembly of synthetic miltiradiene pathways in *S. cerevisiae*, using a fusion of the diterpene synthases *SmCPS1* and *SmKSL1* as well as the fusion of *BTS1* (GGPPS) and *ERG20* (farnesyl diphosphate synthase, FPPS). This approach led to a significant improvement in the production of miltiradiene and a reduction of byproduct accumulation. The miltiradiene yield of the most prolific strain reached 365 mg/L in a 15-L bioreactor culture [82]. Further study revealed that simultaneous overexpression of *tHMGR-upc2.1* and *ERG20-BTS1-SaGGPS* genes had a synergetic effect on the miltiradiene production in yeast strains, and 488 mg/L miltiradiene was produced in fed-batch fermentation [83]. Based on the establishment of miltiradiene production in *S. cerevisiae*, Guo *et al.* engineered the yeast by incorporating modules expressing *CYP76AH1* as well as a P450 reductase gene (*SmCPR1*) into this efficient pathway, leading to the heterologous production of ferruginol at 10.5 mg/L under flask shaking conditions without process optimization [27]. As the biosynthetic pathway of tanshinones remains unsolved and most of the metabolic intermediates could rarely accumulate in plants, the engineered yeast strain provided a basis strain to identify additional enzymes.

The production of RA in microorganisms has also been realized. Bloch *et al*. [84] designed a biosynthetic pathway tailored for the production of RA and isorinic acid (IA) in *Escherichia coli* through the overexpression of a six-enzyme chimeric bacterial and plant pathway. They compared three plant RAS orthologues from *Coleus blumei* (*CbRAS*), *Lavandula angustifolia* (*LaRAS*) and *Melissa officinalis* (*MoRAS*). The strains with different plant RAS genes produced different levels of RA and IA but in the same ratios, and the amount of RA and IA produced could also be increased by the optimization of another pathway. In *S. miltiorrhiza*, the analysis of phenolic acid biosynthesis could provide more candidate modules for pathway optimization. This work provided a microbial platform for the production of RA and IA that uses a modular biosynthetic approach to produce these compounds.

The metabolic engineering of *S. miltiorrhiza* or its hairy roots provides an alternative method for accelerating the production of tanshinones and phenolic acids by overexpression the key enzymes or suppression the competition pathway. *SmGGPPS*, *SmHMGR*, and *SmDXS* were key enzymes involved in the biosynthesis of tanshinone precursors [17,38,48,49,50]. To increase the supply of precursors in *S. miltiorrhiza*, *SmGGPPS1*, *SmHMGR1*, and *SmDXS2* were overexpressed in transgenic hairy root lines. It was revealed that the co-expression of *SmHMGR1* and *SmGGPPS1* resulted in the highest production of tanshinones (approximately 2.727 mg/g dw), which was approximately 4.74-fold higher than the production in the control (0.475 mg/g dw) [17]. The simultaneous overexpression of *SmHMGR* and *SmDXR* in *S. miltiorrhiza* hairy roots also significantly increased the production of tanshinones, especially with elicitor-mediated induction [62].

The production of RA and SAB in *S. miltiorrhiza* hairy root cultures could be greatly increased by overexpression of *SmC4H*, *SmTAT*, and *SmHPPR* or by co-expression of *SmTAT* and *SmHPPR*. Co-expressing *SmTAT1/SmHPPR1* produced the most abundant RA (906 mg/liter) and LAB (992 mg/L) in *S. miltiorrhiza* hairy root cultures, which were 4.3- and 3.2-fold more than in wild-type, respectively [18]. To enrich the precursors available for SAB biosynthesis, Zhang *et al.* simultaneously expressed an *Arabidopsis* *Production of Anthocyanin Pigment 1* transcription factor (*AtPAP1*) and co-suppressed cinnamoyl-CoA reductase (*SmCCR*) and caffeic acid *O*-methyltransferase (*SmCOMT*), two endogenous key enzyme genes, in *S. miltiorrhiza* plants. The accumulation of SAB was up to three-fold higher in these plants than in the control. This was the first study to describe the production of valuable end products through combinational genetic manipulation in *S. miltiorrhiza* plants [85]. The suppression of competing pathways is also an alternative approach for accelerating the conversion of a substrate into the target compound. The suppression of 4-hydroxyphenylpyruvate dioxygenase (*SmHPPD*), which was reported to compete with the biosynthesis of rosmarinic acid, was shown to inhibit the substrate competition from a bypassing pathway, thereby increasing the accumulation of phenolic acids [18].

## 6. Perspectives

Most genes involved in the biosynthesis of tanshinones and phenolic acids in *S. miltiorrhiza* have been cloned either through a homology cloning strategy or by tissue-specific and induced-expression patterns. These genes have been functionally characterized *in vitro* or *in vivo* through enzymatic assays, RNAi, overexpression or isotopic labeling, which provide a basis for investigating the downstream pathways involved in tanshinone and phenolic acid biosynthesis. Firstly, co-expression analysis with the known functional genes could be employed as reference to narrow down the candidate genes obtained from various transcriptomes. In addition, comparative transcriptomes, such as those from different tissues, different development stages, and different induction times, as well as more efficient screening methods, should be applied to offer more information about the candidate genes involved in the biosynthetic pathway. Secondly, obtaining metabolic intermediates involved in biosynthetic pathways is extremely important for the analysis of downstream biosynthesis pathways, and given that the metabolic intermediates are always unstable and accumulated with low level, synthetic biology seems to be an increasingly important approach for analyzing biosynthetic pathways [63]. The functional genes could be incorporated into microorganisms through the MOPE strategy, thereby producing sufficient compounds for use in *in vitro* enzymatic assays. In addition to synthetic biology, the chemical synthesis and exploration of intermediates from plants belonging to the same species are also effective approaches.

The studies on the biosynthetic pathway of tanshinones and phenolic acids in *S. miltiorrhiza* have made significant progress. Tanshinones are widely extant in *Salvia* [7], due to the various biological activities, biosynthetic pathway analysis of tanshinones in *Salvia* will receive increasing attention. 13C tracer and precursor feeding experiments revealed that RA is likely to be the precursor of SAB [15]. The biosynthesis of RA has been well studied in *Coleus blumei* (Lamiaceae) as well as in *S. miltiorrhiza* (Lamiaceae), and the enzymes involved in the biosynthesis of RA have also been elucidated in *Melissa officinalis* (Lamiaceae), *Anchusa officinalis* (Boraginaceae), and *Lithospermum erythrorhizon* (Boraginaceae) [13]. The existing research results for the biosynthesis of tanshinones and RA in *S. miltiorrhiza* have laid a foundation for the analysis of downstream biosynthetic processes, providing more modules for synthetic biology and references for the analysis of the biosynthetic pathways involved in producing phenolic acids and diterpenoids in both *S. miltiorrhiza* and other species.
